# Mesenchymal stem cells in idiopathic pulmonary fibrosis

**DOI:** 10.18632/oncotarget.18126

**Published:** 2017-05-23

**Authors:** Xiaohong Li, Shaojie Yue, Ziqiang Luo

**Affiliations:** ^1^ Department of Physiology, Xiangya School of Medicine, Central South University, Changsha, China; ^2^ Department of Neonatology, Xiangya Hospital, Central South University, Changsha, China

**Keywords:** idiopathic pulmonary fibrosis, mesenchymal stem cells, mobilization, secretome, immunomodulation

## Abstract

Idiopathic pulmonary fibrosis (IPF) is a major cause of respiratory failure in critically ill patients and common outcome of various lung interstitial diseases. Its mortality remains high, and no effective pharmacotherapy, in addition to artificial ventilation and transplantation, exists. As such, the administration of mesenchymal stem or stromal cells (MSCs) is currently investigated as a new therapeutic method for pulmonary fibrosis. Clinical trials on MSC-based therapy as a potential treatment for lung injury and fibrosis are also performed. MSCs can migrate to injured sites and secrete multiple paracrine factors and then regulate endothelial and epithelial permeability, decrease inflammation, enhance tissue repair, and inhibit bacterial growth. In this review, recent studies on stem cells, particularly MSCs, involved in alleviating lung inflammation and fibrosis and their potential MSC-induced mechanisms, including migration and differentiation, soluble factor and extracellular vesicle secretion, and endogenous regulatory functions, were summarized.

## INTRODUCTION

Idiopathic pulmonary fibrosis (IPF) is a chronic, progressive, and irreversible lower respiratory disease characterized by diffuse alveolar inflammation and alveolar structural disorder, and this condition eventually leads to pulmonary interstitial fibrosis [1]. Its main pathological changes include severely damaged alveolar structure, numerous proliferated fibroblasts, and extensive extracellular matrix (ECM) deposition [2, 3]. Pulmonary fibrosis (PF) is a serious respiratory disease whose main clinical manifestation is progressive dyspnea, which results in respiratory failure and death. The incidence of IPF increases annually, its mortality is high, and its median survival is approximately three to five years after diagnosis, but these observations are poorer than those of many cancers [4–6]. However, IPF is quite difficult to diagnose, and no standard treatment is available for patients with IPF [7]. Current treatment mainly involves lung transplantation, mechanical ventilation, and oxygen therapy. Although pirfenidone and nintedanib elicit therapeutic effects on lung functional deterioration and disease progression in patients with IPF, these drugs fail to induce damaged tissue regeneration [8]. As new therapeutic agents for diseases, stem cells, including mesenchymal stem cells (MSCs), have been widely investigated [9]. In phase I clinical trials, MSCs are used as cell therapy for pulmonary diseases because these MSCs, which are a class of multipotent stem cells, can be transdifferentiated, cloned, and self-renewed *in vitro* [10, 11]. MSCs also help ameliorate inflammation and moderate the deterioration of PF [12].

## PATHOLOGIC LESIONS OF IPF

IPF is an interstitial pulmonary disease characterized by dysfunction of epithelial cells, activation of fibroblasts, accumulation of myofibroblasts, and vast deposition of ECM [3]. Fibroblastic foci are important pathological and unique morphological hallmark lesions in IPF, in which fibroblasts and myofibroblasts are possibly involved in tissue remodeling and matrix deposition [13]. The pathologic degree of fibroblast foci is closely related to the prognosis of patients with IPF. Active fibroblasts in PF are formed via at least three mechanisms, namely, proliferating resident fibroblasts, epithelial-to-mesenchymal transition (EMT), and bone marrow (BM)-derived fibrocytes.

### Proliferation of resident fibroblasts

The proliferation and accumulation of resident fibroblasts play a significant role in IPF pathogenesis and constitute a key source of interstitial collagens in fibroblastic foci. Under the action of transforming growth factor-β (TGF-β), resident fibroblasts can be activated and differentiated into myofibroblasts, then accumulating in damaged lung tissues [3]. Intrapulmonary fibroblasts increase the expression of collagen genes and mesenchymal proteins, such as vimentin and α-smooth muscle actin (α-SMA), through Wnt/β-catenin signaling and take part in PF development [14]. Myofibroblasts, which express α-SMA, are the primary inducers of increasing the expression of lung collagen proteins and thus promote ECM deposition and the contractility of lung tissue [15].

### Epithelial-mesenchymal transition

Alveolar epithelial cell (AEC)-derived fibroblasts are another component in fibroblastic foci during PF through EMT, which involves sustained missing of epithelial markers, including E-cadherin, keratin, and continuously increased expression of mesenchymal markers, including N-cadherin, vimentin, α-SMA [16–20]. The establishment of EMT is also implicated in the interaction of TGF-β with receptor tyrosine kinase (RTK) by activating the Ras/ERK/MAPK signaling pathway [17, 21, 22]. TGF-β is a key factor in EMT process during PF development. A previous study provided direct evidence for the involvement of TGF-β in EMT process during PF by generating transgenic mice, in which type II AECs were labeled with β-galactosidase (β-gal) [16]. In the PF model of overexpressing TGF-β1, fibroblasts positive for vimentin were mostly β-gal-positive cells [16]. However, phenotypic changes are fully reversible after inducing factors are removed [18], and EMT contributing to lung fibrosis *in vivo* remains controversial [23]. Endothelial cells of pulmonary blood vessels are one of the major cell types of structural cells and implicated in maintaining homeostasis in lungs [24]. *In vitro* studies have reported that endothelial cells may act as a source of α-SMA-positive mesenchymal cells and can produce type I collagen (Col I) [25, 26]. Hashimoto et al. demonstrated that endothelial cells can stimulate the production of a large number of fibroblasts in bleomycin (BLM)-induced PF model, and the underlying mechanism of EMT in endothelial cells is involved in Ras and TGF-β activation [27].

### Bone marrow-derived fibrocytes

Experimental data have provided evidence that some fibroblasts in fibroblast foci can be derived from BM progenitor cells (BMPCs). The circulating peripheral blood-derived fibroblasts (called “fibrocytes”) have fibroblast-like properties and express CD45^+^ collagen I^+^ CXCR4^+^ [28, 29]. BM-derived fibrocytes can be chemotactically gathered to damaged lung tissue sites and play a key role in the establishment of fibrosis at the injured sites [28, 30, 31]. Clinical examination showed that fibrocytes increased in peripheral blood, bronchoalveolar lavage fluid (BALF), and lung tissues of IPF patients, and this phenomenon was associated with poor patient prognosis [32, 33]. Animal experiments also showed that lung fibrocytes began to increase on the second day after intratracheal administration of BLM, peaking on the eighth day, and still significantly higher than that of the control group until the 20th day [28]. The homing of circulating fibrocytes to fibrotic lung is dependent on the CXCL12/CXCR4 biological axis. Treatment of mice with CXCL12 antibody or CXCR4 antagonist with BLM-induced lung injury inhibited circulating fibrocytes from migrating to the damaged lung tissues and significantly attenuated lung fibrosis [28, 34–36]. Some studies indicated that mouse fibrocytes to traffic to lung via the CCL12/CCR2 axis in the FITC-induced PF model [37, 38] and via the CCL3/CCR5 axis in BLM-induced PF [39].

## ESSENTIAL PROPERTIES OF MSCS

**Figure 1:**
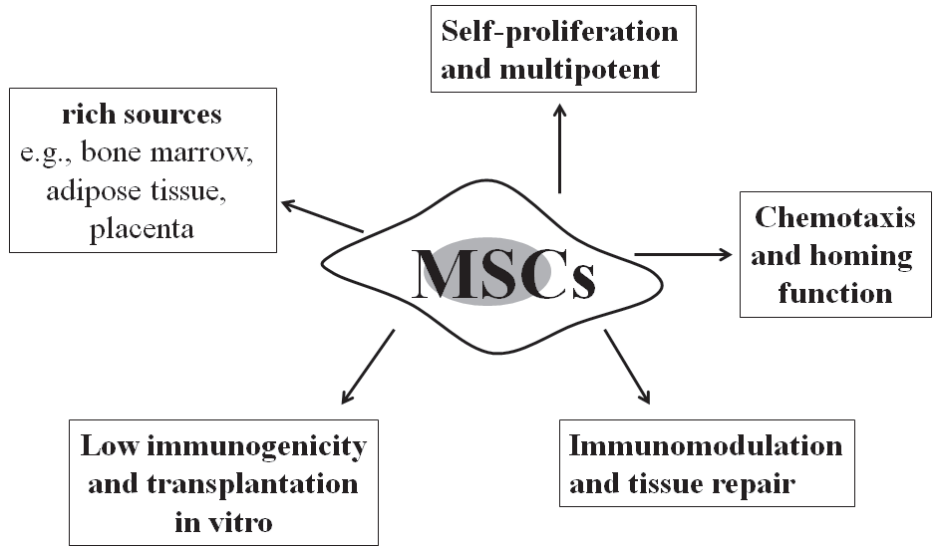
Properties of mesenchymal stem cells Mesenchymal stem cells (MSCs), as a class of multipotent stem cells, possess multiple properties: (i) self-proliferation and differentiation potential; (ii) rich sources, e.g., bone marrow, adipose tissue, and placenta; (iii) low immunogenicity and transplantation *in vitro*; (iv) chemotaxis and homing function; and (v) immunomodulation and tissue repair. These characteristics are crucial for MSCs to be applied as a therapeutic modality in idiopathic pulmonary fibrosis (IPF).

BM can also generate mesenchymal stem cells (bone marrow-derived mesenchymal stem cells, BM-MSCs), which have protective effects against the PF. Friedenstein et al. first discovered MSCs, which are a class of multipotent stem cells with self-proliferation and differentiation potential, in 1968 [40, 41]. MSCs can be obtained from different types of tissues, such as BM, adipose tissue, and umbilical cord blood, and BM-MSCs are the main sources for stem cell therapy [40]. Three criteria have been established for isolating and cultivating MSCs [42–44]: i) MSCs exhibit fibroblastic morphology, clonogene, and plastic adherence when cultured in standard tissue culture conditions; ii) differentiate into adipocytes, osteoblasts, and chondrocytes *in vitro*; and iii) express certain cell surface markers such as CD44, Sca-1, CD29, and CD90 but not CD45, CD34, CD14, and CD11b. Moreover, MSCs possess low immunogenicity, can be used for xenogenous transplantation, and are widely used in basic research and treatment of many diseases due to its immunomodulation and tissue repair function [45] (Figure 1). With further research development, MSC transplantation is increasingly used in the treatment of clinical diseases, such as lung diseases [45], hepatic failure [46], myocardial infarction [47], diabetes [48], sepsis [49], and acute renal failure [50].

Exogenous MSC transplantation has achieved the desired effects, and no transplant-related adverse reaction has been observed in lung-injury animals in some small clinical trials. However, its safety and efficacy in humans remain questionable due to the difference between animals and human and the small sample size of clinical trials. Concurrently, transplant-related adverse reactions have been observed in patients under clinical trials. In clinical research in which BM-MSCs were used for treating osteogenesis imperfecta (OI), one of six children patients who had undergone standard BM transplantation for severe OI had clinically significant toxicity [51]. Moreover, MSC-based treatments pose other risks, such as tumorigenesis due to weakened immune system and enhanced angiogenesis [52].

In recent years, research on the mobilization of endogenous stem cells from BM for tissue repair has attracted people's attention [53, 54]. The BM acts as a repository for a variety of stem cell populations, which are mobilized at varying degrees into the peripheral circulation after damage [55]. Endogenous MSCs from BM can be mobilized and have the capability to proceed from the circulatory system to various, experimental, and damaged tissues, where they can promote to tissue repair and regeneration by secreting various paracrine factors and directly differentiating into different cells [54–57]. Research showed that approximately two EPCs exist in 10^7^ mononuclear cells [58], whereas 0.5-5 MSCs exist in 10^5^ mononuclear cells [59]. Rojas et al. reported that busulfan inhibited BM function before duplicating PF and aggravated the degree of PF in mice model [60]. The administration of *N*-acetylcysteine-pretreated human embryonic MSCs protects against BLM-induced lung injury [61]. Oncostatin M-preconditioned MSCs alleviated BLM-induced PF through paracrine effects of the hepatocyte growth factor (HGF) [62]. These results imply that normal BM function and/or trying to improve MSCs function play important roles in lung injury repair. Granulocyte colony-stimulating factor (G-CSF) promotes stem cell mobilization via downregulating the expression of stromal cell-derived factor (SDF)-1 and increasing CXCR4 in BM [63]. Studies found that transplantation of BM stem cells as well as mobilization by G-CSF promotes recovery after spinal cord injury in rats [64]. G-CSF had a protective effect against BLM-induced lung injury and fibrosis [65]. In newborn rat model of high-oxygen induced-lung injury, G-CSF had a protective effect for alveolar growth restriction caused by high oxygen and improved the serum vascular endothelial growth factor (VEGF) level and promoted lung blood vessel growth [66]. However, other research showed that G-CSF enhanced BLM-induced lung toxicity through a mechanism that probably involved neutrophils [67], and serum concentrations of G-CSF was significantly higher in IPF patients than that of the control group [68]. Meanwhile, the number of BM-MSCs decreased in BLM induced-PF mice model [69]. SDF-1-TR1 and CXCR4 mRNA expressions were significantly increased in BM-MSCs of IPF patients compared with that of controls [70]. The protective effects of BM-derived mononuclear cells from donors of acute respiratory distress syndrome markedly decreased [71]. These results suggest that BM function diminishes in the development of lung injury.

## ROLES AND MECHANISMS OF MSCS IN PULMONARY FIBROSIS

MSCs are also used to treat PF [9]. BM-MSC transplant significantly reduced lung injury and fibrosis in the animal BLM-induced PF models [72]. In 2003, Ortiz reported that BM-MSC injection (5×10^5^/mouse in 200 μl of PBS) through the jugular vein immediately after challenge with BLM can significantly reduce PF [73]. In SiO_2_-induced IPF mice model, human mesenchymal stem cell (hMSC) transplantation directly replaced fibrosis with normal lung cells and reduced IPF symptom, such as collagen deposition and inflammation [74]. BLM-induced lung injury and fibrosis were significantly reduced by injection of BM-MSCs by downregulating proinflammatory and angiogenic cytokines and nitric oxide metabolites after 4 days of BLM inhalation. [75]. Zhao et al. also proved the therapeutic effects of BM-MSC engraftment in BLM-induced lung damage in rats [76]. The cyclophosphamide alone did not improve PF and may even aggravate PF, but the combination with BM-MSCs can protect against BLM-induced lung fibrosis in mice [77]. Moreover, data from MSC-based clinical trials support the safety of a single infusion of hMSC in patients with IPF [78].

### Homing and migration

IPF is an epithelial-driven disease [79]. The recurrent injury and abnormal repair of AECs disturb normal epithelial-fibroblast interactions and play major roles in promoting the fibrotic process [80]. When type I AECs are damaged and/or missing, type II AECs experience hyperplastic proliferation and differentiation into type I AECs to blanket the uncoated basilar membranes [3]. Under pathologic conditions, the resident fibroblasts accumulate and differentiate into myofibroblasts under the action of TGF-β in these damaged areas.

**Figure 2:**
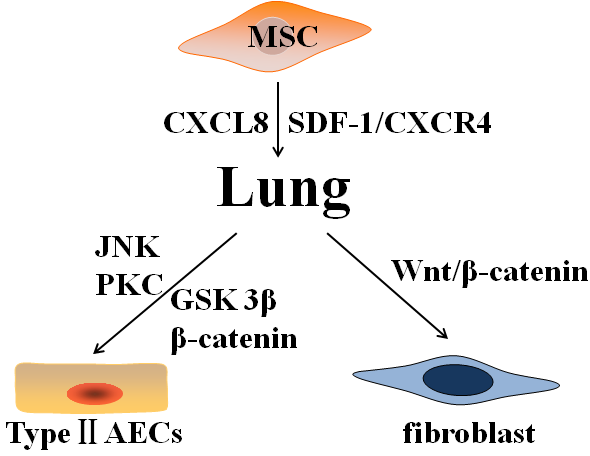
Various differentiations of mesenchymal stem cells after homing to the injured lung Mesenchymal stem cells (MSCs) home to lung in response to injury via chemokines and receptors (CXCL8, SDF-1, and CXCR4). On one hand, MSCs differentiate into type II AECs and ameliorate pulmonary fibrosis through canonical Wnt pathway (GSK-3β and β-catenin) and noncanonical Wnt pathway (JNK and PKC). On the other hand, MSCs differentiate into fibroblasts and promote pulmonary fibrosis by activating Wnt/β-catenin signaling. Abbreviations: AECs: alveolar epithelial cells; CXCR4: C-X-C chemokine receptor type 4; CXCL8: interleukin-8; SDF-1: stromal cell derived factor-1; JNK: c-Jun N-terminal kinase; PKC: protein kinase C; GSK: glycogen synthase kinase.

Some results suggested that BM-MSCs was homing to the lungs after damage, exhibiting epithelioid phenotypes and reducing inflammation and collagen deposition in BLM-induced animal models [73, 81]. Akram et al. found that hMSCs showed a strong migratory response to AECs injury in a 3D direct-contact wound repair model [82]. The migration of BM-MSCs are mediated by some chemotactic factors and their receptors. The chemokine SDF-1 is crucial for migration to injured tissues via interacting with its cognate receptor CXCR4 on the cellular surface [83]. Xu et al. found SDF-1 significantly promoted the chemotactic migration of BM-MSCs, but this effect was mimicked by lungs extracts from mice after BLM treatment and was completely inhibited by a synthetic specific CXCR4 antagonist, that is, TN14003 [36]. SDF-1 and CXCR4 were increased in lungs of IPF patients compared to normal human lungs, and the concentration of SDF-1 in serum and BALF and the expression level of CXCR4 in lungs were elevated in BLM-induced animal models [36]. On day 7 after BLM challenge, the SDF-1α mRNA levels in the lungs increased significantly compared with saline groups and remained on day 14 [29]. SDF-1 expression was also increased in the lungs of patients with idiopathic interstitial pneumonia [84]. Another study showed chemokine CXCL8 (interleukin-8) also promoted the migration of hMSCs [85] (Figure 2).

### Differentiation

Furthermore, after homing to injured lungs, MSCs can differentiate into type II AECs and be involved in the renewal of the alveolar epithelium *in vitro* and *in vivo* [86–88]. MSC differentiation into type II AECs is mainly mediated by the Wnt pathway [89]. Liu et al. found that β-catenin and glycogen synthase kinase-3β (GSK-3β) in the canonical Wnt pathway were activated during the differentiation of mouse MSCs into type II AECs [86]. Overexpression of β-catenin in mouse MSCs to activate canonical Wnt/β-catenin pathway further improved their protective effect against epithelial impairment and therapeutic effects for ARDS in mice [87]. Further studies indicated that Wnt5a contributes to MSC differentiation into type II AECs through noncanonical c-Jun N-terminal kinase (JNK) or protein kinase C (PKC) signaling *in vitro* [88] (Figure 2).

However, MSCs playing a role in resistance to PF through differentiation into epithelial cells remains controversial. In HCl-induced ALI models, MSCs did not improve the pathologic changes of ALI and PF [90]. The researchers found that the activation of canonical Wnt/β-catenin signaling induced most MSCs to differentiate into fibroblasts or myofibroblasts, and that block this signal after MSC transplantation ameliorated PF and improved pulmonary function *in vivo*. Tang et al. also showed that BM-MSCs induce α-SMA-positive myofibroblasts in a transplanted BM model [91]. MSCs, which were administered to mice during the fibrotic stage of radiation-induced PF model, differentiated into fibroblast-like phenotype and aggravated the fibrotic lesion [92]. MSCs isolated from BLM-injured mice lungs were also more likely to differentiate into fibroblasts *in vitro* [93] (Figure 2).

### MSC-derived secretome

**Figure 3:**
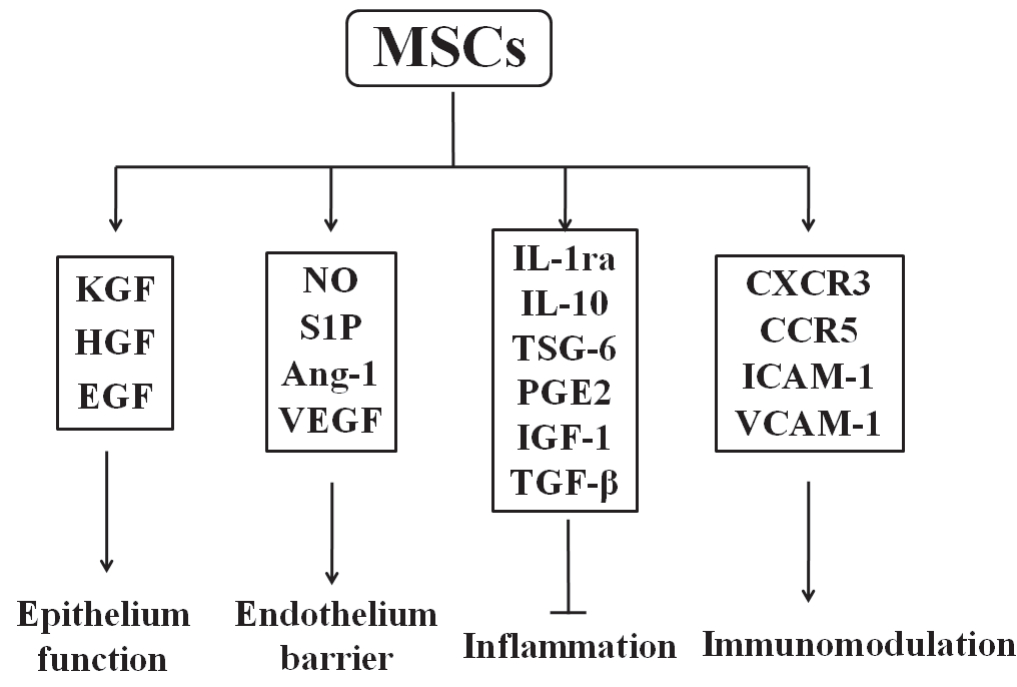
Secretome of mesenchymal stem cells After activation, mesenchymal stem cells (MSCs) can produce various growth factors (KGF, HGF, and EGF) to protect epithelium function. MSCs enhance endothelium barrier via NO, S1P, Ang-1, and VEGF and secrete anti-inflammatory cytokines, such as IL-1ra, IL-10, and TSG-6. Moreover, MSCs achieve immunomodulating function through chemokines and receptors (ICAM-1, VCAM-1, and CXCR3). These factors are crucial components for function regulation and tissue repair by MSCs. Abbreviations: KGF: keratinocyte growth factor; HGF: hepatocyte growth factor; EGF: epidermal growth factor; NO: nitric oxide; S1P: sphingosine-1-phosphate; Ang-1: angiopoietin-1; VEGF: vascular endothelial growth factor; TSG-6: TNF-α stimulated gene/protein 6; PGE2: prostaglandin E2; IL-1ra: IL-1 receptor antagonist; IL-10: interleukin-10; IGF-1: insulin like growth factor 1; TGF-β: transforming growth factor-β; ICAM-1: intercellular adhesion molecule-1; VCAM-1: vascular cell adhesion molecule-1.

MSCs with therapeutic effects on injured lungs have been extensively investigated because of their low engraftment and differentiation after exogenous administration [94, 95]. Although MSCs can migrate to the damaged lung tissues and have differentiation function, these roles are insignificant. The mechanism of MSC resistance to PF mainly depends on the function of their paracrine factors and immune adjustment. Interestingly, MSC-derived conditioned medium (MSC-CM) can also exert their protective effects against BLM-induced lung injury and fibrosis [96]. In the BLM-induced rat model, MSC-CM was proved to prevent PF because of reducing pulmonary inflammation, fibrosis score, collagen deposition, and cell apoptosis [96]. MSC-CM protected A549, namely human non-small-cell lung cancer epithelial cells, from BLM-induced apoptosis [96]. At present, one emerging concept is MSCs having paracrine roles in lung injury repair and regeneration. MSCs have been proposed to possess the capacity to secrete a broad range of bioactive molecules, such as growth factors, cytokines, and chemokines [97–99]. These bioactive molecules regulate local immune response to establish a regenerative microenvironment and subsequently inhibit inflammation and repair the injured tissues (Figure 3).

#### Growth factors

MSC-derived growth factors play essential roles in the repair of alveolar epithelial cells and pulmonary vascular endothelial cells and restoration or maintenance of lung permeability following injury [100]. Keratinocyte growth factor (KGF), the seventh member of the FGF family (FGF7), is an important epithelial-specific growth factor secreted by BM-MSCs [101]. KGF can decrease pulmonary edema, the expression of TGF-β and platelet-derived growth factor-BB (PDGF-BB), and the loss of type II AEC in BLM-induced PF [102]. BM stem cells expressing KGF via an inducible lentivirus protects against BLM-induced PF [103]. Intratracheal administration of MSC-CM one hour following injury decreased inflammation and prevented the influx of neutrophils and pulmonary edema by restoring lung protein permeability and increasing alveolar fluid absorption in the injured alveolus; however, blocking KGF expression by using a neutralizing antibody abrogated their therapeutic properties [101]. Another epithelial-specific growth factors secreted by MSCs are HGF and epidermal growth factor (EGF) [100]. MSCs prerteated with hypoxia had better therapeutic effects in BLM-induced PF and improved the survival rate of transplanted MSCs because of increasing HGF in part [104]. Oncostatin M strengthened the anti-lung fibrosis effect of MSCs through paracrine HGF [62]. Moreover, HGF gene knockdown in the MSCs significantly diminished the protective effects of MSCs on the injured lung, indicating that MSCs restored lung injury by maintaining HGF levels in the lung, and the HGF expression is required for MSCs to protect the injured lung [105]. BM-derived progenitor cells enhance endothelial junction integrity and endothelial barrier function to prevent the increase in pulmonary microvascular permeability and edema formation in mice following LPS challenge by paracrine sphingosine-1-phosphate (S1P) release and activation of Rac-1 and Cdc42 [106]. Angiopoietin-1 (Ang-1) is a known endothelial survival and vascular stability factor that reduces endothelial permeability and suppresses leukocyte-endothelium interactions by modifying adhesion molecules and cell junctions of endothelial cells [107]. MSCs promote therapeutic effects in injured mice by secreting Ang-1 [108]. Ang-1 was responsible for the beneficial effect of MSCs by preventing the formation of actin stress fiber and claudin 18 disorganization through NF-κB suppression [109]. MSCs directly reduced the nuclear translocation of NF-κB in pigs with acute lung injury induced by intravenous oleic acid [110]. VEGF secreted by MSCs was important to maintain alveolar endothelium barrier [111].

#### 3.3.2. Anti-inflammatory cytokines

One important pathogenesis of IPF is acute and/or chronic inflammation, which is also a key factor leading to the majority of IPF patients encountering recurrent lung injury [79]. Initially, IPF is known as an inflammatory-driven disease associated with the interactions of mononuclear cells, fibroblasts and cytokines. In BLM-induced PF models, the early acute inflammation is also important for the onset and progression of late PF [112]. Studies that used MSCs to treat inflammatory lung diseases reported that MSCs possess important anti-inflammation effects, promoting lung tissue repair. For example, some researchers conducted phase I clinical trials to test the safety of MSCs on ARDS patients [113, 114]. Zheng et al. reported the safety of intravenous administration of hMSCs in 12 patients with ARDS in a double-blind randomized single-center trial [115]. MSC transplantation relieved pulmonary inflammation and damage in both intravenous LPS/zymosan-induced extrapulmonary ALI and intratracheal LPS-induced intrapulmonary ALI [116]. MSC transplantation combined with appropriate antimicrobial therapy also obviously decreased the mortality of septic mice through downregulation of inflammation and inflammation-related genes and upregulation the expression of genes for enhancing bacterial clearance [117].

**Figure 4:**
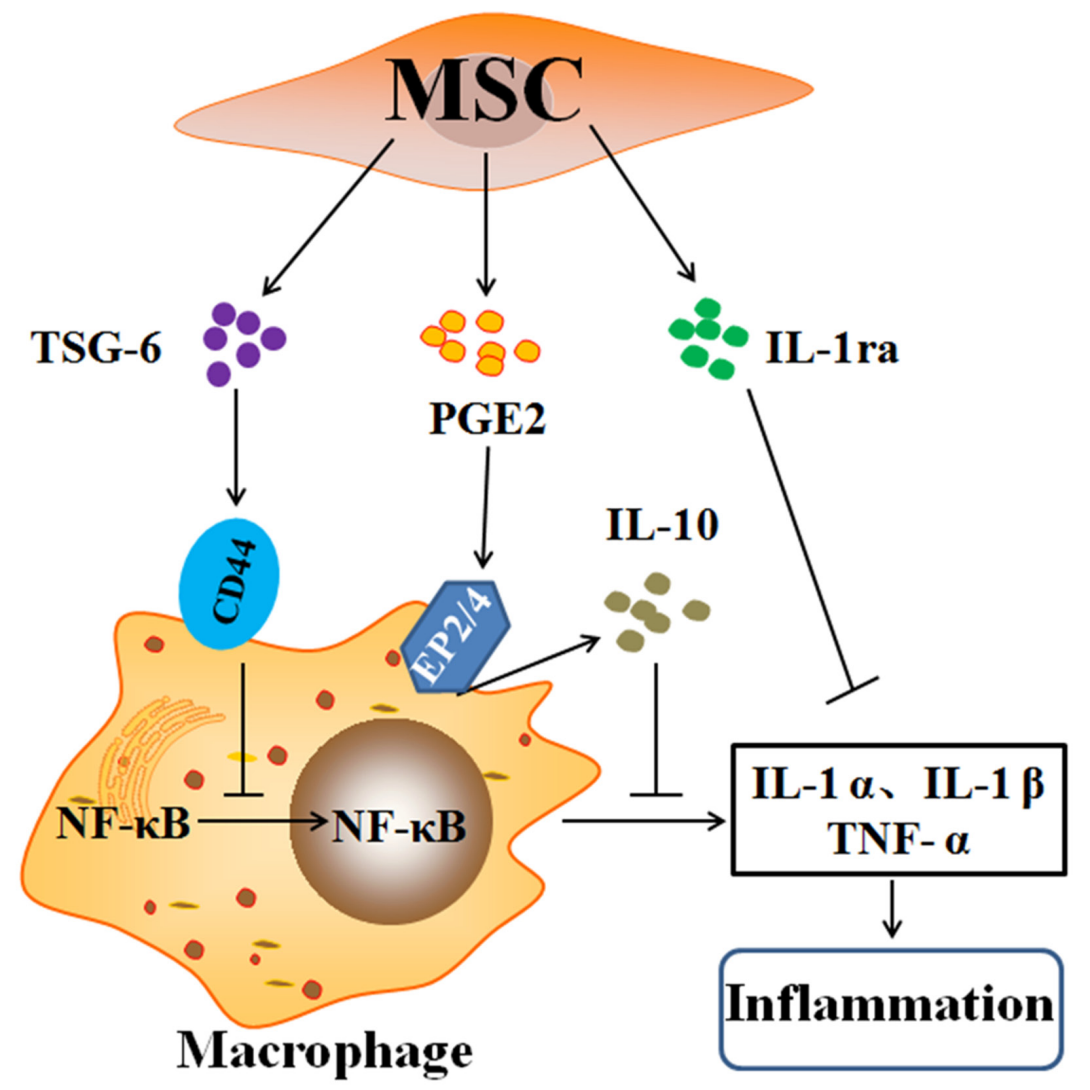
Anti-inflammatory factors secreted by mesenchymal stem cells The anti-inflammatory effects of the mesenchymal stem cells (MSCs) are largely explained by the cells being activated to secrete TSG-6, PGE2, and IL-1ra on resident macrophages. TSG-6 suppressing the activation and translocation of the (NF-κB) complex to the nucleus and decreasing the secretion of the pro-inflammatory cytokines, namely, TNF-α, IL-1α, and IL-1β, is dependent on CD44 expression. PGE2 bind to EP2 and EP4 receptors on macrophages and change macrophages to the phenotype that secretes IL-10. IL-1ra secreted from MSCs inhibit the production of TNF-α through IL-1α-activated macrophages. Abbreviations: TNF-α: tumor necrosis factor-α; IL: interleukin; TSG-6: TNF-α stimulated gene/protein 6; PGE2: prostaglandin E2; IL-1ra: IL-1 receptor antagonist; NF-κB: nuclear factor-κB.

MSCs can secrete cytokine modulators and contribute to their anti-inflammatory effects [118]. In a comparative multiplex analysis, MSC-CM attenuated lung inflammation and promoted an anti-inflammatory M2 macrophage phenotype via insulin-like growth factor 1 (IGF-1) secretion in LPS-induced lung injury [119]. MSC-CM attenuated the influx of inflammatory cells within the alveolar space while reversing histological evidence of lung fibrosis through the restoration of MSCs in the lungs accompanied by the inhibition of T cell proliferation [120]. BM-MSCs decreasing the inflammatory response and preventing the lungs from developing fibrosis are attributed to cell activation to secrete interleukin-1 receptor antagonist (IL-1ra) [121]. Yagi et al. found that hMSCs attenuated systemic inflammation in mice after i.p. injection of LPS through secreting soluble receptor-1 for TNF (sTNFR1), which binds to TNF-α and eliminates its action [122]. Lee et al. suggested that the alleviations of PF animals and IPF people after i.v. injection of MSCs in the tail were caused by MSCs activation to secrete TNF-α-stimulated gene/protein 6 (TSG-6), which is an significant anti-inflammatory gene and expressed in various kinds of cells [47, 123]. However, hMSCs with TSG-6 siRNA were ineffective, and the advantages of hMSCs were greatly magnified through i.v. administration of recombinant human TSG-6 (rhTSG-6) [118, 124]. hMSCs and rhTSG-6 suppressed the Toll-like receptor 2/NF-κB signaling in the lung resident macrophages via directly interacting with CD44, then decreasing the produce of proinflammatory cytokines, TNF-α, and IL-1α [118, 125]. Jarvinen et al. demonstrated that BM-MSCs inhibited T cells activation by secreting prostaglandin E2 (PGE2), thereby stimulating alveolar macrophages secreting IL-10 [126]. PGE2 protects C57BL/6 mice lungs from BLM-induced PF and lung dysfunction [127]. The anti-inflammatory effects of PGE2 secreted by MSCs were dependent on the interaction of EP2 and EP4 receptors on macrophages [49, 118, 128]. Overexpression of EP2 receptor significantly enhanced MSCs migrating to injured lung tissue and further reduced LPS-induced pulmonary vascular permeability by decreasing the levels of proinflammatory cytokines [129]. MSCs transformed classically activated macrophages (M1MΦ) into alternatively activated macrophages (M2MΦ), upregulating IL-10 and IL-6 [130–132] (Figure 4).

However, MSCs ameliorated lung inflammation and fibrosis only when they were injected during the early stage of BLM-induced PF. If the fibrotic changes already exist, then MSCs do not have beneficial effects, and even worsen the lung injury and fibrosis. When MSCs were administered through tail vein on 7 day after BLM challenge, the inflammation had not been improved compared with the BLM model group [82]. It suggests MSCs play protective roles in the development of PF via influencing the early inflammatory process.

#### Immunomodulation

Multiple studies have determined that the production and deposition of extracellular matrix proteins in the process of PF are involved in various pathways, including growth factors, cytokines, and chemokines, associated with inflammation, cell transport, angiogenesis, and immunomodulation. Among these pathways, the role of humoral autoimmunity in IPF is an emerging subject of investigation. Autoantibodies are present in the plasma of >80% of IPF patients [133]. Autoreactive T-cells against lung antigens have been detected in the same studies. IPF patients show peripheral wastage of NK cells and imbalance of the Treg/Th17 axis [134]. A total of 40%-60% of IPF patients may display an autoimmune response against type V collagen [col(V)] [135]. Anti-col(V) immunity is an important factor in IPF pathogenesis, and col(V)-induced tolerance downregulates TGF-β-related signaling pathways and eliminates BLM-induced fibrogenesis [136]. A phase 1 study has been conducted to test the safety and study the effective application of IW001 in the treatment of col(V) antibody-positive IPF patients [137]. Patients with IPF often have elevated serum levels of SP-A and SP-D, which possess significant innate immune function because of the capabilities to give rise to both pro-inflammatory and anti-inflammatory responses [138–140]. SP-D deficiency increased the numbers of macrophages and fibrocytes in lung tissues, the expression of profibrotic cytokines (TGF-β1 and PDGF-AA) in BLM-induced PF. And intratracheal injection of SP-D relieved BLM-induced PF in SP-D^-/-^ mice [141]. These studies support the hypothesis that autoimmune response to autoantibodies may play a vital role in the progression of disease for patients with IPF.

A number of studies have shown that MSC has a strong immunosuppressive effect through producing the abovementioned paracrine factors. However, the mechanisms by which MSCs mediate the immunosuppression are yet to be determined. MSCs produced a large number of chemokines (CXCR3 and CCR5 ligand chemokines) and adhesion molecules, including intercellular adhesion molecule (ICAM)-1 and vascular cell adhesion molecule (VCAM)-1[142, 143]. Then, immune cells aggregated near MSCs, which secreted high concentrations of nitric oxide (NO) and indoleamine-2,3-dioxygenase (IDO) then suppressed T cells proliferation [144]. High concentrations of NO inhibited immune responses by suppressing phosphorylation of signal transducer and activator of transcription (STAT)5 and promoting apoptosis of T cells [145]. Furthermore, MSCs secreted IL-6 *in vitro*, then induced B lymphocytes to produce IgG [146]. A research suggested that the IL-6-dependent PGE2 production played a crucial role in the beneficial therapy of MSCs in experimental mouse arthritis model [147]. However, the expression of cytokine and chemokine receptors was decreased in aged BM-MSCs and their protective roles were compromised due to impaired migration and anti-inflammatory response [148]. This result may be because aging can lead to significant alterations in extracellular matrix composition, inflammatory mediators, and chemokines and increased susceptibility to oxidative stress.

Interestingly, research has shown that MSCs also can secrete TGF-β, which is a key profibrotic protein [149]. This MSC mechanism may explain why they aggravate BLM-induced lung fibrosis when administered to mice during the late fibrotic phase. This result is seemingly contradictory to the antifibrotic effects of MSCs. However, Liu et al. found that the supernatant derived from human BM-MSCs isolated from normal individuals expressed a high level of TGF-β1 and had a better therapeutic effect in reducing the mortality, inflammation, and fibrosis than that from MSCs derived from umbilical cord which secreted a low level of TGF-β1 [150]. In that study, TGF-β1 hypersecretion in BM-MSCs activated IL-6/STAT3 signal pathway, then promoting Tregs proliferation and production of antifibrotic chemokine IFN-γ-inducible protein 10. This study implies that TGF-β secreted by MSCs can modulate immune responses to ameliorate inflammation and fibrosis, and this mechanism may be important to MSC resistance to PF. Moreover, TGF-β1 can regulate immune homeostasis. T cells were abnormally activated and some proinflammatory cytokines were significantly increased in TGF-β1 deficiency mice [151]. IL-4 and/or IL-13 activated the STAT6 signal in BM-MSCs, leading to an increase in TGF-β production, which was useful alone or in combination with regulatory T cells [149]. In the presence of pro-inflammatory cytokines such as IL-1β, IL-6, and IL-23, MSCs retained the ability to inhibit allogeneic T cell proliferation and secreted high levels of TGF-β and low levels of IL-4 [152]. Therefore, the timing of MSC administration is crucial to determine whether the cells will eventually have a favorable or adverse impact on PF development. The greatest benefit of MSCs occurs in the early inflammatory stages of PF.

### Extracellular vesicles

Extracellular vesicles (EVs) are diminutive and orbicular membrane segments, including exosomes, microvesicles (MVs), and apoptotic bodies, as described by the recommendations of the International Society for Extracellular Vesicles [153]. EVs can be produced by various type cells as well as separated from body fluids *in vivo*, involving in cell-to-cell communication and mediating the phenotypic changes of receptor cells [154, 155].

Notably, a number of studies showed that the paracrine benefits of MSCs were also mediated by EVs contained in MSC-CM except the abovementioned soluble factors [97]. Therefore, some researchers proposed that MSC-CM and/or MSC EVs can be used as treatment for acute lung injury and other inflammatory lung diseases. Recently, a research group found that MSCs secreted three types of EVs [156]. MSC-derived EVs also expressed MSC phenotypic markers, such as CD29, CD73, CD44, and CD105, and can be identified through conventional flow cytometry [157]. The microvesicles isolated from MSC-CM reduced the total cell count in BALF, alveolar macrophages ratio, inflammation, and fibrosis [158].

MSC-derived EVs can be an important tool for the clinical benefit of MSC treatment and may reduce risks associated with engraftment of MSCs [159]. Recently, Pachhler et al. proposed a good manufacturing practice-grade standard protocol for exclusively human MSC-derived EVs [160]. The characterization and establishment of MSC-derived EVs will help identify active components in therapeutic EVs for future clinical applications.

MSC-derived extracellular vesicles may be an important tool for the clinical benefit of MSC treatment and may reduce the risk associated with transplanting MSCs.

### Signal molecules regulated by MSCs

MSCs elicit their beneficial effects not only via these mechanisms but also via regulation of some signal molecules to improve the endogenous capability of lungs to resist damage. MSCs reduce the expression of matrix metalloproteinase (MMP9), tissue inhibitor of metalloproteinase-1, γ-interferon, and TGF-β1 to suppress lung inflammation and fibrosis [161]. MSCs also alleviate pulmonary damage and mortality in association with a reduce in macrophage inflammatory protein-2 and TNF-α levels in BALF and high levels of IL-10 in plasma and BALF [162]. Ni et al. suggested that BM-MSCs significantly ameliorate the BLM-induced PF by increasing the gene expression levels of NAD(P)H: quinine oxidoreductase 1, gamma-glutamylcysteine synthetase, heme oxygenase-1, and nuclear factor erythroid 2-related factor 2 [163]. These results indicate that MSCs can promote lung resistance against inflammation and oxidative stress by regulating anti-inflammatory and antioxidant factors.

Scientists are also actively looking for the endogenous mediators induced by MSCs for pulmonary homeostasis. The increasing expression of FoxM1 induced by BMPC treatment is a critical endogenous mediator against inflammatory lung injury [164]. Moreover, The endothelial FoxM1 is required for paracrine released by MSCs in S1P-mediated enhancement of endothelial barrier function [164]. MSCs were susceptible to oxidative stress after decreasing stanniocalcin, whereas stanniocalcin overexpression in MSCs alleviated oxidative stress [165]. STC1 plasmid transfected to MSCs enhanced the capability of MSCs to ameliorate the fibrosis by reducing oxidative stress, endoplasmic reticulum stress, and TGF-β1 production in AECs [165]. MSCs expressing stanniocalcin 2 (STC2) exhibit increased cell viability, improved cell survival, and increased pluripotency and self-renewal marker expression by activating p-AKT and p-ERK1/2 signal pathways under oxidative conditions [166]. Ahmad et al. proved that MSCs overexpressing the mitochondrial transport protein Miro1 promoted mitochondrial transfer from MSCs to damaged epithelial cells and rescued the epithelial injury [167]. These results suggest that FoxM1, stanniocalcin, and Miro1 may be essential factors that regulate the endogenous injury repair and mediate the effects of MSCs to ameliorate lung injury and PF.

## CONCLUSION AND PERSPECTIVES

In summary, MSC administration can be an effective therapy to alleviate BLM-induced lung injury and fibrosis. The mechanisms involve multiple biological effects of MSCs, including homing, differentiation, secretome, and promotion of lung endogenous antidamage ability. However, a large proportion of studies have explore the early inflammatory stage rather than the late fibrotic stage. This is a major limitation that MSC administration is only used as a preventive measure but not as a treatment modality. Therefore, the timing of MSC administration is crucial. Some controversies on the effectiveness and safety of MSC administration for IPF have been presented. Thus, further research on the mobilization of endogenous MSCs from BM and its mechanisms should be performed. Recently, MicroRNAs are found to participate in IPF pathogenesis [168]. However, the role of MicroRNAs in improving the beneficial effects of MSCs is unknown. Hence, further research and clinical trials should be conducted.
